# Stroke and plasma markers of milk fat intake – a prospective nested case-control study

**DOI:** 10.1186/1475-2891-8-21

**Published:** 2009-05-21

**Authors:** Eva Warensjö, Annika Smedman, Birgitta Stegmayr, Göran Hallmans, Lars Weinehall, Bengt Vessby, Ingegerd Johansson

**Affiliations:** 1 Department of Public Health and Caring Sciences, Clinical nutrition and metabolism, Uppsala University, Uppsala, Sweden; 2 Smart Foods Centre, University of Wollongong, NSW, Australia; 3 Department of Public Health and Clinical Medicine, Umeå University, Umeå, Sweden; 4 National Board of Health and Welfare, Stockholm, Sweden; 5 Department of Odontology, Umeå University, Umeå, Sweden

## Abstract

**Background:**

Dairy products are high in saturated fat and are traditionally a risk factor for vascular diseases. The fatty acids 15:0 and 17:0 of plasma lipids are biomarkers of milk fat intake. The aim of the present study was to evaluate the risk of a first-ever stroke in relation to the plasma milk fat biomarkers.

**Methods:**

A prospective case-control study was nested within two population based health surveys in Northern Sweden. Among 129 stroke cases and 257 matched controls, plasma samples for fatty acid analyses were available in 108 cases and 216 control subjects. Proportions of 15:0 and 17:0 of plasma lipids, weight, height, blood lipids, blood pressures, and lifestyle data were employed in conditional logistic regression modelling.

**Results:**

The proportions of fatty acids 17:0 and 15:0+17:0 of total plasma phospholipids were significantly higher in female controls than cases, but not in men. 17:0 and 15:0+17:0 were significantly and inversely related to stroke in the whole study sample as well as in women. The standardised odds ratio (95% CI) in women to have a stroke was 0.41 (0.24–0.69) for 17:0 in plasma phospholipids. Adjustment for traditional cardiovascular risk factors, physical activity and diet had marginal effects on the odds ratios. A similar, but non-significant, trend was seen in men.

**Conclusion:**

It is hypothesised that dairy or milk fat intake may be inversely related to the risk of a first event of stroke. The intriguing results of this study should be interpreted with caution. Follow up studies with greater power, and where intakes are monitored both by dietary recordings and fatty acid markers are needed.

## Background

Stroke is a major cause of disability and death in the developed world, and it is predicted to become an increasing health threat in developing countries too [1]. Many risk factors have been linked to development of vascular diseases including intake of fat and dietary fatty acid profile [2,3]. Thus, a diet high in saturated fatty acids and serum cholesterol has traditionally been considered a risk for vascular diseases [4]. As milk and dairy products contribute to saturated fat intake in many populations an association between vascular diseases and consumption of dairy products was suggested [5]. Results from ecological studies supported this hypothesis [6].

However, in the latest decades seemingly contradictory results have been found in several cohort studies, suggesting that dairy products, some compound(s) of dairy products, or life style factors associated with dairy product intake may be related to a reduced risk for heart disease and stroke [7-10].

Exposure to dairy products may be directly estimated by dietary recording methods and/or indirectly by using biomarkers. These two principal approaches benefit from not being associated with the same sources of error [11]. Intake measures in general, and fatty foods in special, are prone to underreporting, and measured intakes of energy, fat, and fatty foods correlates moderately with intakes estimated by other dietary registration methods [12-14]. Biomarkers, which are unrelated to systematic and random recall bias in diet recordings, may add beneficial information. Pentadecanoic (15:0) and heptadecanoic (17:0) acid content in plasma/serum and erythrocyte membranes have been shown to be markers for milk fat intake [15-18].

In northern Sweden the prevalence of cardiovascular diseases has been among the highest in the country [19]. Trends in disease incidence and risk factors are studied in the repeated Northern Sweden MONICA (Monitoring of Trends and Determinants in Cardiovascular disease) screenings [20], and the Västerbotten Intervention Programme [21]. In both projects lifestyle information and blood is collected and stored to allow for prospective, e.g. diet-disease, studies. The aim of the present study was to prospectively evaluate the risk of a first-ever stroke event in participants in the MONICA and VIP studies in relation to estimated milk fat intake mirrored as the proportion of fatty acids 15:0 and 17:0 in plasma phospholipid and cholesteryl ester fractions, respectively.

## Methods

### Study population

In 1985 a community intervention programme for the prevention of cardiovascular disease and diabetes was initiated in the county Västerbotten, Northern Sweden called the Västerbotten Intervention Programme (VIP). At the same time the Northern Sweden MONICA centres were set up. The health survey part of the VIP and MONICA studies, which were essentially similar, has been described in detail elsewhere[21,22]. Between 1^st ^January 1995 and 31^st ^December 1996 more than 40,000 men and women had participated in the VIP study. For each MONICA survey (1986, 1990, 1994) a total of 2,000 individuals were invited and altogether 4,725 individuals participated [22]. The present study is a case-control study nested within these two cohorts. The study was approved by the ethics committee at Umeå University, and the participants of both the MONICA and VIP surveys consented to donate a blood sample to the Northern Sweden Medical Research Bank for future research.

### Case finding

The case finding of stroke was based on mainly three sources; discharge records from hospitals, reports from general practitioners and death certificates. When found the cases were registered in a standardized way using the MONICA manual [23]. The examination used to define the stroke cases are described elsewhere [24]. Special emphasis was placed on uniform case ascertainment and diagnostic criteria throughout the project. For the present study, cases were defined between 1^st ^January 1985 and 31^st ^December 1996. During this time 166 individuals, who had participated in the VIP or MONICA study and donated a blood sample to the blood bank, suffered from a first ever ischemic, haemorrhagic or unspecified stroke before the age of 75. Individuals with a prior acute myocardial infarction (n = 15) or a cancer diagnosis (n = 13) were excluded from the study. Also, nine cases with a previous stroke event were excluded. Thus, 129 stroke cases (46 women and 83 men) remained for analyses (101 ischemic, 22 haemorrhagic, and 6 unspecified). Two controls were randomly selected from the MONICA and VIP cohorts for each case and matched for gender, age (± 2 years), date and type of health survey, and living region. In one set only one control was matched.

### Biomedical analyses

Participants were asked to fill out a questionnaire, including information on health status, physical activity, tobacco use and diet. Questions on physical activity at work and leisure time, and tobacco use (none, daily, or ex-smoking or snuff use, respectively, were answered. Food intake frequencies were reported in a semi-quantitative FFQ as: never, some time per year, 1–3 times per month, once every week, 2–3 times per week, 4–6 times per week, once every day, 2–3 times per day, 4 times per day or more. Intakes per day were then calculated as previously reported [16]. For the present study dietary data were not available in all individuals since optically readable FFQs were not available in the early phases of the basic surveys. Further, early non-optically readable FFQs were not identical between health centres. The analyses involved dietary data from the optically readable FFQ, and aggregates of questions (food groups) in selected sections where the FFQ questions were identical.

Blood pressure was recorded either in a supine or recumbent position after a five minute rest as previously described [25]. Blood samples for lipid measurements were obtained after at least 4 hours of fasting. Total cholesterol was measured using a Reflotron bench top analyser or an enzymatic method (both from Boehringer Mannheim GmbH Diagnostica, Germany). Plasma glucose (after at least 4 hours of fasting and 2 hours after an oral glucose challenge) was analysed using a Reflotron bench top analyser as described previously [25]. Body weight and height were measured when the participant wore light clothes but no shoes. BMI was calculated as weight (kg)/height (m^2^).

### Fatty acid analyses

The fatty acid composition of the serum lipids was analyzed as described in detail elsewhere [26]. Briefly, serum lipids were extracted in chloroform and the different lipid fractions (phospholipids and cholesteryl esters) were separated by thin-layer chromatography, and then fatty acids were transmethylated. The fatty acid methyl esters were separated with gas chromatography using a Hewlett Packard GLC system consisting of a HP 5890 Series II GC apparatus, HP 7673 automatic sampler, HP 3365A Series II Chemstation integrator software and a 50 m × 0.25 mm CP-Sil 88 Chrompack capillary column, with helium as carrying gas. Standards from Nu Check (Elysian, Missouri, USA) were used for identification of individual fatty acids and as a control of the GC system. The proportions of fatty acids are given as the relative percentage of the sum of the fatty acids analyzed. Fatty acids analysed were 14:0, 15:0, 16:0, 16:1 n-7, 17:0, 18:0 n-9, 18:1, 18:2 n-6, 18:3 n-6, 18:3 n-3, 20:3 n-6, 20:4 n-6, 20:5 n-3, 22:5 n-3 and 22:6 n-3. The coefficients of variations for the analyses were <10% for all fatty acids in both fractions, except for 15:0 in cholesteryl esters which was slightly higher (13.4%). Plasma samples for fatty acid analyses were available for 66 cases and 134 controls in men and 42 cases and 82 controls in women.

### Statistical Analysis

The statistical analyses were carried out with the software package STATA, version 10.0 (STATA Corp LP, College Station, Texas, USA). Analyses were run on all participants as well as on gender strata. The distribution of continuous variableXs was evaluated with Shapiro Wilk's test. Not normally distributed variables were log-transformed to improve normality. Descriptive measures for continuous variables are presented as mean with SD for normally distributed variables and for non-normally variables as median with interquartile rage. Differences in baseline characteristics between cases and controls were tested with Student's t-test or Mann Whitney's depending on distribution. A *P*-value of < 0.05 was considered statistically significant. Odds ratios with 95% confidence intervals (CI) to have a first ever stroke were calculated using conditional logistic regression. The percentages of 15:0, 17:0 and 15.0+17:0 in the phospholipid and cholesteryl ester fractions were mean centered and univariance standardized before entered into the models as continuous variables. Physical activity (leisure time and occupational) was used a categorical variable graded 1–5 (1 for lowest activity), as were smoking and snuff use, i.e. no use (coded 0), ex-use (coded 1) and present use (coded 2). Food groups were treated as continuous variables as servings per day. Crude models and models adjusted for various possible confounders, such as BMI, serum cholesterol, smoking, systolic and diastolic blood pressure, physical activity, tobacco use and intakes of fish, fruits and berries, vegetables and alcohol were carried out. Wald's test was used to identify non-influential variables in the different models.

## Results

The stroke events occurred on average 36 months after the participation in the health surveys [25]. Baseline characteristics for cases-controls with plasma for fatty acid analysis stratified by gender are presented in Additional file 1. Female cases had higher systolic blood pressure and male cases had both higher systolic and diastolic blood pressures than their respective controls. Similar results were obtained when baseline characteristics for all cases (n = 129) and controls (n = 257) were evaluated (data not shown). In both genders ischemic stroke was the most common type of stroke. Thus, in women 90.5% (n = 38) of all stroke cases were ischemic, 7% (n = 3) intracerebral haemorrhagic stroke, and one case was unspecified (2.5%). In men 74% (n = 49) were ischemic stroke, 23% (n = 15) were intracerebral haemorrhagic stroke, and 3% (n = 2) were unspecified stroke.

The proportion of 17:0 and 15:0+17:0 in plasma phospholipids were significantly higher in female controls than female cases (p = 0.001, Additional file 1). On average the relative proportion of 17:0 was 10% higher in the controls than cases. The proportion of 15:0 in plasma phospholipids and 15:0 and 17:0 in plasma cholesteryl esters followed the same trend, but the difference did not reach statistical significance (Additional file 1). No significant differences were seen in men for any of these variables, but controls had numerically higher mean levels of 17:0 and 15:0+17:0 in plasma phospholipids than cases (Additional file 1).

In women and men taken together as well as in only women, all biomarker fatty acids in the phospholipid fraction were inversely related to a first ever stroke. Similarly to 15:0 in women, 15:0, 17:0 and 15:0+17:0 in phospholipids in men rendered odds ratios less than 1.0, but did not reach statistical significance Adjustment for BMI, s-cholesterol, tobacco use, and systolic and diastolic blood pressure in the model regressing either milk fat biomarker in the phospholipid fraction strengthened the relationship marginally in women, but weakened the odds ratio marginally in men (Table 1).

**Table 1:** Odds ratios (95% CI) to have a first ever stroke (any type) using mean centered, univariance standardized values for fatty acids 15:0, 17:0 and 15:0+17:0 proportions of the plasma phospholipid fraction in separate models.

	Model	15:0 (phospholipid %)	17:0 (phospholipid %)	15:0+17:0 (phospholipid %)
All	Crude	0.81 (0.62–1.1)	0.67(0.51–0.89)	0.70(0.53–0.92)
	Model 1	0.81(0.60–1.1)	0.71(0.52–0.97)	0.72(0.52–0.99)
Men	Crude	0.86 (0.62–1.2)	0.88 (0.63–1.2)	0.86 (0.61–1.2)
	Model 1	0.92(0.63–1.3)	0.95(0.66–1.4)	0.93(0.64–1.4)
Women	Crude	0.73 (0.48–1.1)	0.41 (0.24–0.69)	0.48 (0.29–0.79)
	Model 1	0.56(0.31–1.0)	0.37(0.18–0.74)	0.41(0.22–0.78)

Model 1: BMI, serum-cholesterol, tobacco use, systolic blood pressure, diastolic blood pressure

When examining the association of 17:0 in phospholipids with stroke in women, adjustment for most food items, alcohol and physical activity had virtually no effect the odds ratios (Table 2).

**Table 2:** Odds ratios (95% CI) to have a first ever stroke (any type) using mean centered, univariance standardized values of the 17:0 proportion of the plasma phospholipid fraction when standardizing for possible biological and dietary confounders

	**Women**	
	
Variables included in adjusted models	n-values	Odds ratio (95% CI)
BMI, s-cholesterol, tobacco use SBP, DBP^1^	111	0.37 (0.18–0.74)
Physical Activity (work and leisure)	66	0.42 (0.20–0.90)
Fish (lean and fatty types)^2^	116	0. 40 (0.23–0.71)
Fruits^2^	115	0.42 (0.24–0.72)
Vegetables^2^	66	0.42 (0.19–0.92)
Alcohol^2^	75	0.43 (0.22–0.85)

^1^s- for serum, and SBP and DBP for systolic and diastolic blood pressure, respectively.

^2^Foods were entered into the models as servings per day for fruits (berries, apples, bananas and citrus fruits), vegetables (white cabbage, root beets, tomatoes, cucumber, spinach and frozen vegetables, and alcohol (wine, beer and spirits). For varying n-values see the Material and Methods section. Models employing the optically readable FFQs (n = 66) followed the same trend as data presented in the Table.

## Discussion

Recent studies on the association between dairy fat intake and vascular diseases are seemingly contradictory to the traditional hypothesis that a high intake of dairy products is a risk factor for such diseases. Thus, studies from Scandinavia reported an inverse association between the estimated intake of milk fat, mirrored as fatty acid biomarkers, and coronary heart disease [27,28] as well as a lower risk of diabetes [29]. Further, an inverse relationship was reported between stroke and dairy intake recorded by a 7-day food diary in the Caerphilly cohort (UK) of elderly men [10]. In the present study, which is the first study investigating the association between the risk of a first stroke and estimated milk fat intake mirrored as plasma 15:0 and 17:0 status, confirmed an inverse association in men and women taken together for the proportion of 17:0 in the plasma phospholipid fraction. This association was driven by a significant and inverse association in women. The trend was similar, but non-significant, for 15:0 in women, and for 15:0 and 17:0 in men. That 17:0 in phospholipids, but not in cholesteryl esters, were inversely related to the risk of stroke may be due to the lower proportion of this fatty acid in the cholesteryl ester fraction.

In the present study, the baseline characteristics of the controls are in accordance with those previously reported in the population in Northern Sweden [21], as well as in the entire study sample. The population based prospective design and that the study sample is representative of the population are strengths of the study. Other strengths are the careful and systematic diagnosis of stroke, and that the fatty acid analyses were performed at a highly renowned laboratory. The weaknesses of the study include the comparably small number of cases and that dietary registrations were only available in a subsample of the study group. This led to that the number of investigated case-control sets went down when adjusting for food groups. However, repeating the crude analysis on the individuals with complete dietary data rendered similar odds ratios, but with slightly wider 95% CIs.

In the present study the relative proportion of 17:0 and 15:0+17:0 in phospholipids were significantly higher in female controls than stroke cases. The odds ratio to have a first ever stroke was 0.41 with 95% CI of 0.24–0.69 for each SD increase of 17:0 in the plasma phospholipid fraction. A similar, but non-significant, trend was seen in men (OR = 0.88, 95% CI 0.63–1.2). Adjustment for blood lipid status, blood pressure, physical activity, and food groups affected the odds marginally in women. In adjusted analyses (Table 2) the odds of a first ever stroke decreased with about 60% for each SD increase in the proportion of 17:0. We have no explanation for the gender difference, but it may reflect gender differences in preferences among various dairy products, or other life style factors. One study has investigated the trends in food intake (1986–1999) in the Northern Sweden study population from which the present study participants were selected. Indeed, women reported a slightly higher intake of cream, sour cream and other more fat dense dairy products as well as a higher intake of solid and soft cheeses. Intakes of milk, sour milk and yogurt products were comparable between men and women [30]. This may have influenced the findings in the present study.

The inverse relationship between estimated intake of milk fat and stroke might seem paradoxical, since milk fat is high in saturated fatty acids. However, studies on the relationship between dietary fat and stroke are sparse and contradictory [31]. In the health professional follow up study (725 stroke cases during 14 years) no relationship was found between stroke and total fat, type of fat, or high fat dairy products [32]. In the Framingham heart study, the risk of ischemic stroke in men decreased across increasing quintiles of total, saturated and monounsaturated fat [33]. In contrast, Wiberg et al [3] reported baseline proportions of 16:0, 16:1 and 18:1 in cholesteryl esters (reflecting saturated fat intake) to be positively and the proportion of 18:2 n-6 in cholesteryl esters (reflecting dietary polyunsaturated fatty acid linoleic acid) inversely related to stroke and transient ischemic attacks.

Assuming dairy products have an inverse association with stroke, several components in milk may candidate for positive causal effects. Calcium, potassium, and magnesium are present in dairy products. These micronutrients may mediate beneficial effects on several risk factors for stroke, e.g. blood pressure, insulin resistance, platelet aggregation and atherosclerotic processes [34]. In the Nurses' Health Study intake of calcium, potassium, and magnesium was each inversely associated with ischemic stroke, but not other types of stroke, and a stronger association was seen with dairy than for non-dairy calcium intake [35]. However, in the Honolulu Heart Program study in elderly men, with a low calcium intake, total calcium intake was unrelated, but calcium from dairy sources inversely associated, with having a stroke [36]. Some dairy products are low in sodium, which may be beneficial for blood pressure [34]. Further, indirect support that milk fat per se may affect stroke risk factors is gained from a cross-sectional study where high fat dairy consumption was inversely related to risk factors, for example blood pressures, insulin, waist and triacylglycerol, while low-fat dairy consumption was not [37]. However, in the present study the inverse relationship between the milkfat biomarkers and a first ever stroke was not affected by blood pressures and other risk factors, ruling out those factors as possible mediators. However, it is possible that micronutrients and other factors found in dairy products may be related to the development of stroke by other mechanisms than adjusted for in our models.

It is also possible that exposure to different dairy products may affect the risk of stroke in different ways. In a recent study cream intake was inversely related to ischemic stroke, while intakes of other dairy products were not [38]. Since a previous study from the same population study as ours [30] indicated that women had a higher intake of more fat dense dairy products, this [30] and the former study [38] indirectly supports the results in the present study. Moreover, in a prospective Scottish study the risk of death from stroke was inversely but non-significantly related to regular consumption of milk [39].

An eventual "milk effect" may also reflect intakes of other nutrients and/or a healthier behaviour in general, but adjustment for various lifestyle factors, i.e. physical activity and vegetables, fruits, fish and alcohol intake did not affect the "protective" effect.

## Conclusion

In the present study we report a higher proportion of the milk fat biomarker 17:0 in the phospholipid fraction in plasma to be inversely related to the risk of a first event of stroke. The trend was similar in the entire study sample and in both genders, but statistical significance was only reached in the entire study sample and in women. The present finding is intriguing, but should be interpreted with caution for various reasons, including that (*i*) the proportion of the milk fat biomarker in cholesteryl ester and phospholipid fractions did not follow the same pattern, (*ii*) the association was only significant in women, and (*iii*) the association with the level of the milk fat marker fatty acid 15:0 deviated from that of fatty acid 17:0. At present we hypothesize that estimated milk fat intake is associated with a lower risk of first event stroke, but this should be confirmed in studies with greater power, where intakes are monitored both by dietary recordings and fatty acid markers.

## Competing interests

None of the authors report any conflict of interest. AS is now an employee of the Swedish Dairy Association. However, the study was carried out before AS started her employment at the association. EW has received compensation for speaking engagements from the Swedish Dairy Association and the International Dairy Federation. AS has received compensation for speaking engagements from the Swedish Dairy Association.

## Authors' contributions

EW carried out the statistical analyses and drafted the manuscript, AS helped drafting the manuscript, BS is the principal investigator of the stroke cohort, GH is the principal investigator of the Northern Sweden Medical Research Bank, LW is responsible for the VIP cohort, BV supervised the fatty acid analyses, and IJ is the principal investigator of the Northern Sweden Diet Database. Everyone took part in the interpretation of the results, writing and finalising the manuscript.

## Supplementary Material

Additional file 1 **Baseline characteristics by gender and case-control status in subjects with plasma for fatty acid (FA) analysis**. Describes of baseline characteristics of study participants in the present case-control study. Click here for file
