# Supplementary material for: Stroke and plasma markers of milk fat intake – a prospective nested case-control study
Source: Nutr J. 2009 May 21;8:21. doi: 10.1186/1475-2891-8-21 (PMC2689251; doi:10.1186/1475-2891-8-21)
Supplement: Additional file 1 — Baseline characteristics by gender and case-control status in subjects with plasma for fatty acid (FA) analysis. Describes of baseline characteristics of study participants in the present case-control study. [file 1475-2891-8-21-S1.doc]

**Additional file 1**: Baseline characteristics by gender and case-control status in subjects with plasma for fatty acid (FA) analysis.

| **Characteristics** | **Women** | | | **Men** | | | **All** | | |
| --- | --- | --- | --- | --- | --- | --- | --- | --- | --- |
|  | Case  (n=42) | Control  (n=82) | *p*- value | Case  (n=66) | Control  (n=134) | *p*- value | Case  (n=108) | Control  (n=216) | *p*- value |
|  | Mean (SD) | Mean (SD) |  | Mean (SD) | Mean (SD) |  | Mean (SD) | Mean (SD) |  |
| Age (years)^1^ | 60 (50-60) | 60 (50-60) | NS | 59.5 (50-60) | 60 (50-60) | NS | 60 (50-60) | 60 (50-60) | NS |
| Fasting p-glucose  (mmol/l)^1,2^ | 5.3 (4.9-5.9) | 4.7 (5.2-5.5) | NS | 5.5 (5.0-5.7) | 5.3 (4.8-5.8) | NS | 5.4 (5.0-5.8) | 5.2 (4.8-5.7) | 0.01 |
| 2 hour p-glucose  (mmol/l)^2^ | 7.6 (2.9) | 7.3 (2.0) | NS | 6.7 (2.3) | 6.4 (2.1) | NS | 7.1 (2.6) | 6.7 (2.1) | NS |
| BMI (kg/m^2^) | 28.1(4.9) | 27.2 (5.2) | NS | 26.8 (3.8) | 25.8 (2.9) | NS | 27.3 (4.3) | 26.4 (4.0) | NS |
| Total s-cholesterol  (mmol/l)^2^ | 6.8 (1.5) | 6.5 (1.4) | NS | 6.3 (1.2) | 6.0 (1.4) | NS | 6.5 (1.3) | 6.2 (1.4) | NS |
| SBP (mmHg)^3^ | 150 (27) | 140 (22) | 0.02 | 146 (20) | 137 (20) | 0.0001 | 148 (23) | 138 (21) | 0.0000 |
| DBP (mmHg)^3^ | 88 (10) | 84 (11) | NS | 90 (12) | 85 (13) | 0.0007 | 90 (12) | 85 (12) | 0.0002 |
| 15:0 (PL%)^4^ | 0.22 (0.04) | 0.24 (0.05) | 0.09 | 0.23 (0.05) | 0.23 (0.04) | NS | 0.23 (0.05) | 0.23 (0.04) | NS |
| 17:0 (PL %)^4^ | 0.47 (0.08) | 0.52 (0.07) | 0.001 | 0.49 (0.07) | 0.50 (0.07) | NS | 0.49 (0.07) | 0.51 (0.07) | 0.007 |
| 15:0+17:0 (PL %)^4^ | 0.70 (0.1) | 0.75 (0.07) | 0.007 | 0.72 (0.1) | 0.73 (0.1) | NS | 0.71 (0.1) | 0.74 (0.1) | 0.02 |
| 15:0 (CE %)^4^ | 0.30 (0.07) | 0.32 (0.08) | NS | 0.32 (0.09) | 0.32 (0.09) | NS | 0.31 (0.08) | 0.32 (0.08) | NS |
| 17:0 (CE %)^4^ | 0.10 (0.02) | 0.11 (0.02) | NS | 0.11 (0.03) | 0.11 (0.03) | NS | 0.11 (0.03) | 0.11 (0.02) | NS |
| 15:0+17:0 (CE %)^4^ | 0.41(0.07) | 0.42 (0.09) | NS | 0.43(0.1) | 0.43(0.1) | NS | 0.42(0.01) | 0.43(0.01) | NS |

PL phospholipids, CE cholesteryl esters

^1^ Data are given as median (interquartile range) for these variables, but as means (SD) for the remaining variables

^2^s- and p- for serum and plasma content, respectively

^3^SBP and DBP for systolic and diastolic blood pressure, respectively

^4^Percent (%) refers to proportion of total FA content in the phospholipid and cholesteryl ester fractions in plasma, respectively.
